# Exploring the Medicinal Potential of *Taraxacum Kok-Saghyz* (*TKS*) Using Widely Targeted Metabolomics

**DOI:** 10.3390/metabo15050306

**Published:** 2025-05-03

**Authors:** Michele Tan, Jeffrey Shih-Chieh Chu, Daniel Robin Swiger

**Affiliations:** 1 Kultevat Inc., 1100 Corporate Square Drive Suite 261, Creve Coeur, MO 63132, USA; 2 Metware Biotechnology Inc., 8A Henshaw St., Woburn, MA 01801, USA; jeff.chu@metware-bio.com

**Keywords:** *Taraxacum kok-saghyz*, metabolomics, bioactive compounds, flavonoids, secondary metabolites, KEGG pathway analysis, antioxidant potential

## Abstract

**Background/Objectives**: Plant-derived secondary metabolites have long contributed to the discovery of novel therapeutic agents, especially in the treatment of parasitic and infectious diseases in developing countries. Metabolomics provides a systems-level approach to understanding plant biochemistry, enabling the discovery of secondary metabolites with pharmacological relevance. *Taraxacum kok-saghyz* (*TKS*), widely known for its rubber-producing capabilities, remains underexplored as a medicinal plant. Given the well-established therapeutic properties of *Taraxacum officinale* and the emerging pharmacological profiles of related species, this study investigates the metabolic composition of *TKS* roots and leaves to uncover bioactive compounds with antioxidant, anti-inflammatory, or hepatoprotective potential. **Methods:** Widely targeted metabolomics was conducted on 10-month-old field-grown Kultevar™ *TKS* plants using ultra-performance liquid chromatography–tandem mass spectrometry (UPLC-MS/MS). Samples were hand-harvested and preserved on dry ice to maintain biochemical integrity. Metabolite identification and classification were performed using the MWDB and KEGG databases. Principal component analysis (PCA) and orthogonal partial least squares discriminant analysis (OPLS-DA) were used to evaluate metabolic variation between tissues. **Results:** A total of 1813 metabolites were identified, including flavonoids, alkaloids, lipids, amino acids, and phenolic compounds. Differential analysis revealed 964 significantly altered metabolites—609 downregulated and 355 upregulated in roots relative to leaves. Multivariate analysis confirmed clear tissue-specific metabolic profiles. KEGG pathway enrichment highlighted the involvement of flavonoid biosynthesis, amino acid metabolism, and lipid metabolism pathways, suggesting bioactive potential. This study presents the first comprehensive metabolic profile of *TKS*, highlighting its potential value beyond rubber production. The detection of numerous therapeutic secondary metabolites supports its promise as a pharmaceutical and nutraceutical resource. Further functional validation of identified compounds is warranted.

## 1. Introduction

Metabolomics enables a comprehensive evaluation of metabolic processes and specialized compounds in plants, offering critical insights into stress response, biosynthetic regulation, and bioactivity [1,2,3,4]. *Taraxacum kok-saghyz* (*TKS*), also known as Russian dandelion, is traditionally cultivated for its high rubber yield [5] and is emerging as a sustainable alternative to *Hevea brasiliensis* in industrial latex production [5]. However, its medicinal potential remains under-characterized, especially in contrast to the widely studied *Taraxacum officinale*, which contains pharmacologically active constituents such as chicoric acid, taraxasterol, and sesquiterpene lactones [6,7,8].

*T. officinale* and other *Taraxacum* species are rich in flavonoids, phenolic acids, terpenoids, and polysaccharides—classes of compounds shown to exert antioxidant, hepatoprotective, anti-inflammatory, and anticancer effects [8,9,10,11]. Their bioactivity supports immune regulation, metabolic modulation, and neuroprotection. Environmental factors, plant part, and developmental stage all influence metabolite abundance and biological activity [10,11,12,13,14].

Despite growing interest in dandelion species [5,10,15,16], *TKS* has not been fully investigated using high-resolution metabolomics. Advances in UPLC-MS/MS, KEGG-based annotation, and network pharmacology now enable in-depth pathway mapping and the functional interpretation of plant metabolites Studies on related species such as *T. mongolicum* and *T. sinicum* have revealed wide metabolic diversity, including glycosides, alkaloids, and bioactive nanovesicles, which influence gut microbiota, vascular health, and oxidative stress resilience [1,3,8,17,18].

Multivariate modeling tools like principal component analysis (PCA) and orthogonal partial least squares discriminant analysis (OPLS-DA) support metabolite classification and biomarker discovery, even with limited sample sizes [2,19,20,21]. Such approaches have enabled the detection of disease-relevant metabolic shifts and the identification of functional clusters in plant pharmacology [2,3,20,22].

This study applies widely targeted metabolomics to characterize the root and leaf metabolomes of field-grown *TKS* plants. By integrating multivariate statistical modeling with KEGG-based pathway enrichment, we aim to uncover therapeutic compounds in *TKS* that may parallel or complement those found in *T. officinale* [1,3,10,22]. This work expands the known utility of *TKS*, potentially positioning it as a dual-purpose crop for both industrial and medicinal use. Additionally, it contributes to a growing body of literature supporting the application of metabolomics in plant-based drug discovery and functional food development [3,5,22,23].

## 2. Materials and Methods

The Kultevar™ *Taraxacum kok-saghyz* (*TKS*) dandelion plants were cultivated under natural open-field conditions to ensure physiological relevance and optimal growth. Seeds were directly sown in soil, and plants were allowed to reach full maturity—approximately ten months after germination—at which point roots and leaves were fully developed and suitable for metabolomic analysis. To minimize post-harvest metabolic changes, plants were hand-harvested, washed thoroughly to remove soil and debris, and rapidly packed with dry ice for overnight transport. This protocol aligns with best practices in plant metabolomics and is consistent with the sample preservation techniques adopted in prior studies examining phytochemical stability in *Taraxacum* species and other Asteraceae [13,21,24]. 

Ultra-performance liquid chromatography-tandem mass spectrometry (UPLC-MS/MS) analysis was conducted using an ExionLC™ AD UPLC system and a QTRAP^®^ 6500+ mass spectrometer equipped with an electrospray ionization (ESI) source, both manufactured by SCIEX, Framingham, MA, USA. Chromatographic separation was performed using an Agilent SB-C18 column (1.8 µm, 2.1 mm × 100 mm) sourced from Agilent Technologies, Santa Clara, CA, USA. Analytical-grade solvents were used throughout the study. Methanol and acetonitrile (chromatographically pure) were purchased from Thermo Fisher Scientific, Waltham, MA, USA, while formic acid (chromatographically pure) was obtained from Sigma-Aldrich, St. Louis, MO, USA.

Metabolites were extracted from 100 mg of lyophilized plant tissue (root or leaf), following a 70% methanol-based protocol to ensure high metabolite recovery, especially for semi-polar compounds like flavonoids and phenolic acids [8,13,25]. Methanol has been widely validated for its ability to extract a broad range of secondary metabolites in dandelions and other medicinal plants [8,24,25]. Samples were vortexed, sonicated at 40 °C for 30 min and centrifuged at 12,000× *g* for 10 min at 4 °C, and the supernatant filtered through a 0.22 µm membrane filter. Extracts were stored at −80 °C until LC-MS/MS analysis [6,25,26].

Ultra-performance liquid chromatography-tandem mass spectrometry (UPLC-MS/MS) was performed using an Agilent SB-C18 column (1.8 µm, 2.1 mm × 100 mm). The mobile phase included solvent A (0.1% formic acid in water) and solvent B (0.1% formic acid in acetonitrile), operated under a gradient elution program—a protocol shown to provide excellent chromatographic resolution for polyphenols and glycosides in complex plant matrices [8,14,27]. The flowrate was 0.35 mL/min, with the injection volume set to 2 µL. Column temperature was maintained at 40 °C. Detection was carried out on a SCIEX AB6500 QTRAP mass spectrometer equipped with an electrospray ionization (ESI) source in both positive and negative ion modes. Operating parameters were a source temperature of 550 °C, an ion spray voltage pf\\of +5500 V (positive) and −4500 V (negative), curtain gas at 25 psi, GSI at 50 psi, and GSII at 60 psi. Metabolites were quantified using multiple reaction monitoring (MRM), with optimized declustering potentials and collision energies per compound [1,14,17].

Metabolites were identified using Metware Biotechnology Inc.’s in-house metabolomics database (MWDB), based on MS/MS spectra, retention time, isotopic patterns, and mass accuracy. Identified compounds were annotated and mapped to the Kyoto Encyclopedia of Genes and Genomes (KEGG) (accessed on 23 October 2023) database to assess functional pathways and biological significance, as demonstrated in similar metabolomic studies on *Taraxacum mongolicum* and *T. officinale* [1,3,28].

Three biological replicates were analyzed per tissue type (root and leaf), with each replicate consisting of pooled material from multiple plants to reduce intra-group variability—an approach consistent with best practices in plant metabolomics [1,4]. Data analysis employed both principal component analysis (PCA) and orthogonal partial least squares discriminant analysis (OPLS-DA), implemented using the MetaboAnalystR package (v1.0.1) [19]. Prior to analysis, metabolite abundance data were log_2_-transformed and mean-centered [1,18,19].

To evaluate model robustness and avoid overfitting given the limited sample size, 200-iteration permutation testing was conducted, wherein class labels were randomly shuffled to generate null distributions. The resulting OPLS-DA model demonstrated strong performance, indicating high explanatory and predictive power. Although no formal a priori power calculation was conducted, a medium-to-large effect size (Cohen’s *d* ≈ 0.8) was referenced illustratively to contextualize the potential for detecting biological differences in small-*n* studies; this is consistent with previous guidance on multivariate modeling power in small metabolomics datasets, though not originally defined in terms of Cohen’s *d* [2,20,29]. This assumption was not used to guide the study design, but it is supported retrospectively by the observed data: 964 differentially expressed metabolites were identified, many with fold changes ≥2 or ≤0.5 and VIP scores >1.0, indicating substantial group separation. These findings support the capacity of multivariate modeling to detect meaningful biological variation even with small sample sizes, consistent with earlier guidance in metabolomics modeling frameworks [2,20,29].

## 3. Results

The metabolomic analysis of *Taraxacum kok-saghyz* (*TKS*) revealed distinct biochemical compositions between root and leaf tissues, highlighting key metabolic differences. Through targeted and untargeted metabolomic approaches, we identified a diverse range of metabolites, including flavonoids, polyphenols, alkaloids, and lipid-derived compounds. These results provide valuable insights into the functional specialization of *TKS* tissues and their potential for pharmacological or nutraceutical applications.

### 3.1. Metabolite Identification and Classification

A total of 1813 metabolites were identified in *TKS* across five major biochemical classes (Table 1), with 964 of these showing statistically significant differences between root and leaf tissues (Table 2). This differential metabolite distribution underscores the tissue-specific biosynthetic specialization and aligns with findings from other *Taraxacum* species, such as *T. officinale* and *T. mongolicum*, where the distinct partitioning of bioactive compounds has also been observed [15,20,30].

### 3.2. Principal Component Analysis (PCA) and OPLS-DA

**Figure 1:**
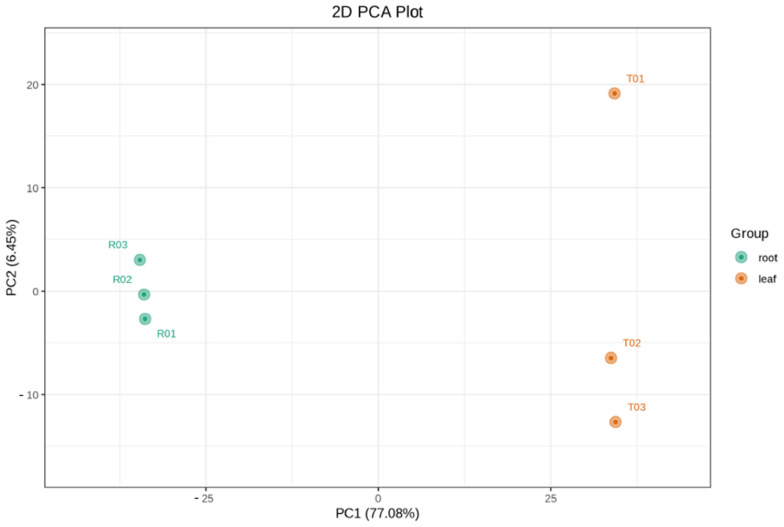
PCA score plot showing clustering of root and leaf samples.

PCA and OPLS-DA analyses were performed to explore tissue-specific metabolic differences between *TKS* leaves and roots. The PCA plot demonstrated a clear separation between leaf and root samples along the first two principal components, which together explained a substantial proportion of the total variance. This clustering pattern reflects distinct biochemical compositions between tissue types, suggesting strong metabolic specialization (Figure 1). OPLS-DA was employed to further discriminate between the two groups and identify key metabolites contributing to the observed differences. The model yielded robust performance metrics (R^2^Y = 0.987, Q^2^ = 0.711), indicating both strong explanatory power and predictive accuracy. Permutation testing validated the model’s reliability by showing that the original model consistently outperformed randomized versions. The Variable Importance in Projection (VIP) scores ranked phenolic compounds, flavonoids, alkaloids, and lipid derivatives among the most discriminatory metabolites.

### 3.3. OPLS-DA and Differential Metabolite Analysis

A total of 964 metabolites were differentially expressed between leaf and root tissues, with 355 upregulated and 609 downregulated in roots relative to leaves (Table 2). This distribution underscores the biosynthetic partitioning of specialized metabolites in *TKS*.

In leaf tissues, several notable secondary metabolites were significantly enriched. 1,3-O-Dicaffeoylquinic acid (cynarin), a polyphenol with antioxidant and hepatoprotective activity, exhibited high relative abundance [23]. Chicoric acid, a caffeic acid derivative with antiviral, antioxidant, and anti-inflammatory properties, was also detected with notable abundance. Furthermore, several flavonoid derivatives—particularly luteolin-7-O-glucoside, orientin, and isoorientin—were present at elevated levels in leaves, aligning with their known roles in oxidative stress mitigation and neuroprotection. A carotenoid-derived compound, (6R,9R)-3-Oxo-α-ionol-β-D-malonyl-glucoside, was also upregulated, potentially contributing to plant defense and signaling.

Root tissues, in contrast, showed higher levels of alkaloids, lipid oxidation products, and quinone derivatives. Among these, (9Z,11E,13E,15Z)-4-Oxo-9,11,13,15-Octadecatetraenoic acid was enriched, indicating lipid peroxidation and potential roles in abiotic stress response. Additionally, 1,2,4-Trihydroxyanthraquinone, a quinone compound with reported antimicrobial activity, was found in greater abundance in roots.

**Figure 2:**
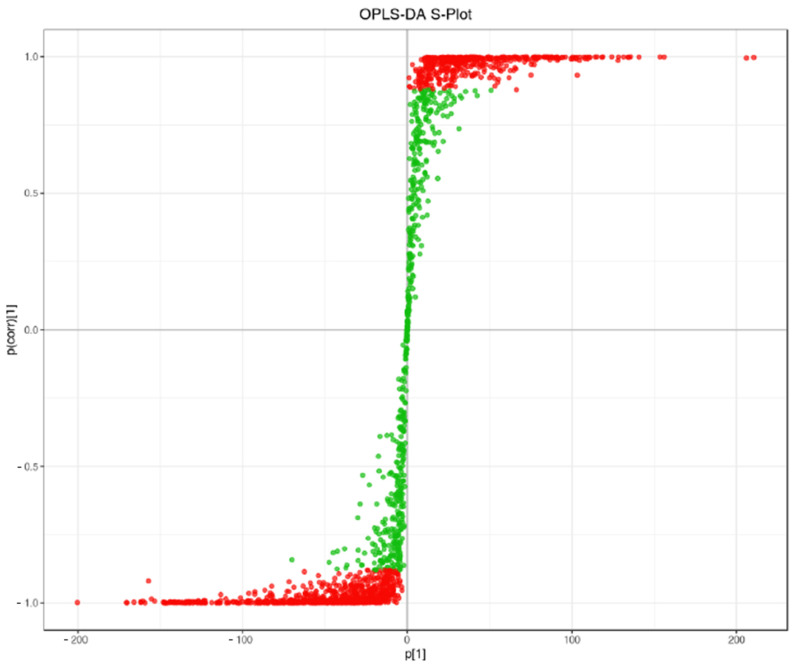
OPLS-DA score plot illustrating metabolic differences between root and leaf samples.

Hierarchical clustering analysis and KEGG-based annotation revealed the tissue-specific enrichment of metabolic pathways. Leaf metabolites were predominantly involved in flavonoid biosynthesis and phenylpropanoid metabolism, while root-specific compounds mapped to lipid metabolism, alkaloid biosynthesis, and quinone pathway. These results support the hypothesis that *TKS* exhibits metabolic compartmentalization akin to other *Taraxacum* species and are further supported by the separation pattern observed in the OPLS-DA S-plot (Figure 2).

The observed biochemical differentiation between leaves and roots has implications for both pharmacological and industrial applications. Leaf-derived compounds show promise for antioxidant and anti-inflammatory uses, while root-enriched metabolites may serve roles in antimicrobial, stress-adaptive, or anticancer formulations.

These findings contribute to the growing understanding of tissue-specific metabolic specialization in Asteraceae plants and highlight the potential of *TKS* as a source of diverse bioactive compounds.

### 3.4. KEGG Pathway Analysis

**Figure 3:**
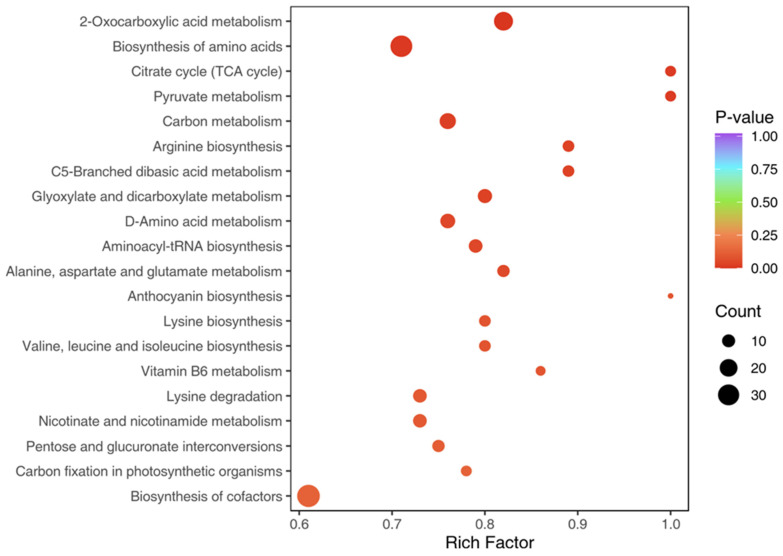
KEGG enrichment diagram of differential metabolites Note: The X-axis represents the Rich Factor and the Y-axis represents the pathway. The color of points reflects the *p*-value. The darker the red, the more significant the enrichment. The size of the dot represents the number of enriched differential metabolites.

KEGG pathway analysis and KEGG enrichment analysis revealed that differential metabolites were involved in primary metabolic pathways such as amino acid biosynthesis, flavonoid biosynthesis, and lipid metabolism (Table 3). These results are further visualized in the KEGG enrichment plot, which illustrates pathway significance and metabolite distribution across biosynthetic routes (Figure 3). The presence of secondary metabolites, including polyphenols and terpenoids, suggests specialized adaptations in *TKS* for stress tolerance and growth regulation. The presence of chicoric acid, a known antioxidant found in *Taraxacum officinale*, suggests potential medicinal applications for *TKS*. Chicoric acid and related compounds suggest antioxidant and hepatoprotective potential [8,9,31,32,33,34,35]. This pathway-level enrichment aligns with the functional potential of leaf-derived metabolites for immunomodulation and root-enriched metabolites for environmental resilience [24,36,37,38].

### 3.5. Medicinal Potential of TKS

*Taraxacum officinale* (common dandelion) is extensively documented for its medicinal properties due to its diverse repertoire of bioactive compounds. Chicoric acid, one of its major phenolic constituents, demonstrates potent antioxidant, antiviral, and anti-inflammatory activities [8,9,39]. The flavonoids luteolin and apigenin, also present in *T. officinale*, are widely studied for their anticancer, antioxidant, and neuroprotective roles. Another important metabolite, taraxasterol, has been shown to possess anti-inflammatory and hepatoprotective effects [7,17,40,41]. Furthermore, sesquiterpene lactones contribute to the plant’s antimicrobial and immunomodulatory potential. In animal models, extracts of *T. officinale* have demonstrated the ability to reduce fatigue and modulate immune responses [11,14,21,42,43].

In the current metabolomic analysis of *Taraxacum kok-saghyz* (*TKS*), 1,3-O-dicaffeoylquinic acid (cynarin)—a polyphenol known for its hepatoprotective and antioxidant properties—was found to be significantly upregulated in leaf tissues [13,32,33]. Chicoric acid was positively identified in the dataset, exhibiting notably high relative abundance in leaf tissues, which supports its therapeutic relevance for antioxidant and hepatoprotective applications [8,9,44]. In addition, several luteolin derivatives were detected in leaf tissues, including luteolin-7-O-glucoside, orientin, and isoorientin, which are associated with antioxidant, anti-inflammatory, and neuroprotective functions [17,45,46]. Conversely, taraxasterol was not detected in either tissue, suggesting its absence or undetectable levels in the examined field-grown *TKS* plants. Despite this, the richness in structurally related phenylpropanoids and flavonoids implies that *TKS* may harbor analogs with comparable pharmacological activity [11,30,31,47].

Our findings mirror established tissue-specific metabolite partitioning patterns observed in *T. officinale* and *T. mongolicum*, where leaves predominantly accumulate polyphenols and flavonoids, while roots are richer in terpenoids, alkaloids, and inulin [10,11,15,35,36,37,38]. In *TKS*, leaf tissues exhibited significantly elevated levels of polyphenolic compounds such as cynarin, chicoric acid, and caffeoyl-glucosides, suggesting antioxidant, immunomodulatory, and hepatoprotective capacities [22,48,49,50,51]. Meanwhile, root tissues revealed higher levels of alkaloids, lipid derivatives, and anthraquinones—metabolite classes implicated in plant defense, stress adaptation, and potential anticancer activity [50,52,53].

This metabolic divergence is consistent with stress-induced reprogramming and biosynthetic specialization documented in related species such as *T. coreanum* and *T. mongolicum*. Under environmental stimuli, these species activate distinct metabolic cascades, and transcriptomic studies implicate SnRK2-ABA signaling and bZIP transcription factors in modulating the biosynthesis of key secondary metabolites, including flavonoids and terpenoids [17,54].

KEGG pathway enrichment in *TKS* not only highlighted key metabolic routes—flavonoid biosynthesis, lipid metabolism, and amino acid metabolism—but also uncovered potentially actionable regulatory nodes for metabolic engineering [35,55,56,57]. Future applications of network pharmacology may leverage these insights to elucidate gene–metabolite relationships and identify candidate biosynthetic genes for bioactive compound enhancement.

The novelty of this study lies in its comprehensive characterization of pharmacologically relevant metabolites [58,59,60] in *TKS*—a species traditionally known for its rubber-producing capabilities [5] but seldom studied for medicinal use. While *TKS* shares a genus with *T. officinale*, it remains largely unexamined in the context of ethnomedicine and functional nutrition. This metabolomic profiling establishes a foundational chemical blueprint that may support the repositioning of *TKS* as a dual-purpose crop for sustainable rubber production and pharmaceutical or nutraceutical applications.

Future research should prioritize the bioassay-guided isolation and functional validation of the identified metabolites through in vitro and in vivo assays. Given the limited ethnopharmacological history of *TKS*, toxicological screening is critical to ensure safety before therapeutic development. Additionally, integrating metabolomic data with transcriptomic and proteomic profiles will aid in uncovering the regulatory networks underpinning tissue-specific metabolite accumulation and support future biotechnological interventions for enhanced metabolite production.

## 4. Discussion

The metabolomic profiling of *Taraxacum kok-saghyz* (*TKS*) revealed a diverse array of metabolites, including flavonoids, alkaloids, lipids, amino acids, and phenolic compounds, with notable differences between roots and leaves. These findings align with the extensive literature on *T. officinale*, *T. mongolicum*, and related species, which are known to contain rich reservoirs of bioactive compounds such as chlorogenic acid, taraxasterol, chicoric acid, and sesquiterpene lactones [58,59,61].

The elevated presence of flavonoids and phenolic compounds in *TKS* leaves suggests antioxidant and anti-inflammatory potential, supported by numerous studies documenting similar properties in *T. officinale* and *T. mongolicum* [55,58,62,63,64]. Furthermore, cardioprotective effects of dandelion preparations have been explored, particularly in the context of vascular health. Phenolic glycosides, dandelion polysaccharides, and related compounds have shown promise for modulating oxidative stress pathways and immune responses [60,64,65,66].

In contrast, the root metabolome of *TKS* exhibited a greater abundance of alkaloids, lipid derivatives, and quinone compounds—typically associated with plant defense, antimicrobial activity, and stress tolerance. These patterns are consistent with the enhanced biosynthesis of bioactive compounds in the roots of *T. mongolicum* and *T. coreanum* under abiotic stress [54,63,67,68].

Principal component analysis (PCA) and orthogonal partial least squares discriminant analysis (OPLS-DA) confirmed the separation of metabolic profiles between tissues, highlighting their specialized biosynthetic roles. The leaf upregulation of cynarin, chicoric acid, and other phenolic compounds supports their value in nutraceutical formulations [58,61,63], while root-specific quinones and oxidized lipids may contribute to environmental adaptation and phytochemical defense [63,68,69].

KEGG pathway analysis revealed that these metabolites are actively involved in flavonoid biosynthesis, lipid metabolism, and amino acid synthesis. These biosynthetic pathways were also highlighted in transcriptomic and proteomic studies across the *Taraxacum* genus. These analytical strategies are supported by optimized sample matching and compound discovery frameworks in high-throughput metabolomics [16,55,61].

Recent work has identified SnRK2 regulation, ABA signaling, and transcriptional control via bZIP proteins as key modulators of metabolite accumulation [17]. Additionally, exosome-like nanovesicles derived from *T. officinale* demonstrated systemic effects on inflammation, metabolism, and vascular function via gut microbiome modulation [40]. Several studies report the hepatoprotective, anti-diabetic, and anticancer properties of dandelion-derived secondary metabolites, highlighting their potential pharmaceutical value [24,31,32,37,56].

This study is the first to comprehensively characterize both root and leaf tissue metabolomes of *TKS* using widely targeted UPLC-MS/MS, expanding its potential beyond rubber production to therapeutic applications. While prior research has demonstrated the bioactivity and health benefits of dandelion-derived compounds in animal models and preclinical contexts [70,71,72], formal toxicological validation remains limited. Recent work has emphasized the agronomic and physiological benefits of plant-based extracts, including dandelion, yet highlighted the lack of dedicated safety assessments for therapeutic use [73]. Moreover, *TKS* lacks a well-established ethnopharmacological record, underscoring the need for cautious and rigorous evaluation prior to medical application [74]. Ensuring safety through comprehensive toxicity testing will therefore be essential before pursuing any in vivo studies or clinical translation.

These findings not only support the phytomedical potential of *TKS* but also contribute to the broader understanding of metabolic plasticity in Asteraceae plants under field conditions. Overall, this study reinforces the view of *TKS* as a metabolically diverse species with significant pharmacological and nutraceutical promise. Future work should aim to isolate and validate key metabolites through bioassays and to compare molecular regulation across *Taraxacum* species to guide targeted applications [74,75,76].

With its dual-use potential and increasing cultivation, *TKS* may serve as a sustainable source of bioactive phytochemicals for next-generation nutraceuticals and plant-derived therapeutics.

## 5. Conclusions

This study provides the first comprehensive metabolomic characterization of *Taraxacum kok-saghyz (TKS)*, highlighting its potential as a medicinal and nutraceutical resource beyond its established role in natural rubber production. Using UPLC-MS/MS-based profiling, we identified 1813 metabolites spanning major classes, including flavonoids, alkaloids, lipids, amino acids, and phenolic compounds. Tissue-specific metabolic differences were clear: leaf tissues were enriched in flavonoids and phenolic acids, while roots exhibited higher levels of lipid-derived metabolites, alkaloids, and anthraquinones. These distribution patterns align with findings in *T. officinale* and *T. mongolicum*, reflecting conserved biosynthetic specialization across the genus.

KEGG pathway enrichment further revealed significant involvement in flavonoid biosynthesis, lipid metabolism, and amino acid synthesis, which are associated with key biological roles in antioxidant defense, immune modulation, and stress adaptation. Notably, compounds such as cynarin and caffeoyl-glucosides detected in *TKS* leaves are linked to hepatoprotective and anti-inflammatory activities, while root-enriched quinones and alkaloids may offer antimicrobial or anticancer benefits.

The novelty of this study lies in repositioning *TKS* as a dual-purpose crop. While most phytochemical studies focus on *T. officinale*, *TKS* remains underexplored despite sharing a metabolite repertoire with recognized therapeutic relevance. By establishing a robust chemical foundation, this work opens avenues for bioassay-guided discovery, pharmaceutical development, and nutraceutical formulation.

Future research should prioritize the in vitro toxicological evaluation of candidate compounds to assess their safety profile before progressing to pharmacological validation. Given the limited ethnopharmacological history of *TKS*, screening for cytotoxicity, genotoxicity, and other potential hazards is critical to ensure safe therapeutic applications. Following these safety assessments, both in vitro and in vivo pharmacological evaluations can be conducted to explore the bioactivity of *TKS* compounds. Additionally, integrative omics approaches, including transcriptomics and network pharmacology, could accelerate the elucidation of biosynthetic regulation and guide the sustainable, dual-use cultivation of *TKS* for both industrial and therapeutic applications.

## Figures and Tables

**Table 1:** Classification and quantification of identified metabolites in *Taraxacum kok-saghyz*.

Metabolite Class	Number Identified
Flavonoids	450
Alkaloids	320
Lipids	275
Amino Acids	190
Phenolic Compounds	578

**Table 2:** Differential metabolite expression between root and leaf Tissues.

Sample Comparison	Total Differential Metabolites	Upregulated	Downregulated
Root vs. Leaf	964	355	609

**Table 3:** KEGG pathway enrichment analysis of identified metabolites.

KEGG Pathway	Number of Metabolites
Amino Acid Biosynthesis	112
Flavonoid Biosynthesis	89
Lipid Metabolism	135

## Data Availability

The data supporting the findings of this study are available upon reasonable request from the corresponding author.

## Abbreviations

The following abbreviations are used in this manuscript:

bZIP	Basic Leucine Zipper Protein
CUR	Curtain Gas
DOAJ	Directory of Open Access Journals
ESI	Electrospray Ionization
GSI	Ion Source Gas I
GSII	Ion Source Gas II
KEGG	Kyoto Encyclopedia of Genes and Genomes
LD	Linear Dichroism
MDPI	Multidisciplinary Digital Publishing Institute
MRM	Multiple Reaction Monitoring
MWDB	Metware Biotechnology Inc. in-house Metabolomics Database
OPLS-DA	Orthogonal Partial Least Squares Discriminant Analysis
PCA	Principal Component Analysis
SnRK2-ABA	SNF1-Related Protein Kinase 2-Abscisic Acid
*TKS*	*Taraxacum kok-saghyz*
UPLC-MS/MS	Ultra-Performance Liquid Chromatography-Tandem Mass Spectrometry
VIP	Variable Importance in Projection
